# NASA’s InSight mission on Mars—first glimpses of the planet’s interior from seismology

**DOI:** 10.1038/s41467-020-15251-7

**Published:** 2020-03-19

**Authors:** Brigitte Knapmeyer-Endrun, Taichi Kawamura

**Affiliations:** 1 Bensberg Observatory, University of Cologne, Cologne, Germany; 2 Université de Paris, Institut de physique du globe de Paris, CNRS, Paris, France

**Keywords:** Planetary science, Physics

## Abstract

NASA’s InSight mission is the first lander to deploy a seismometer on a planetary body since more than 40 years. With a year of seismic data from Mars, new discoveries on Mars’ tectonics and interior structure are just emerging.

## Aiming at the heart of the planet

Unlike all previous Mars missions, which explored the geology, chemistry, and atmosphere of the red planet in great detail, the **In**terior exploration using **S**eismic **I**nvestigations, **G**eodesy and **H**eat **T**ransport (InSight) mission is focused on the interior structure and processes of Mars. It is high time to address these topics as our knowledge of the Martian interior—or, in fact, that of any other terrestrial planet—is poorer than our knowledge for the Earth was 100 years ago. While it is generally assumed that differentiation processes early in the formation of terrestrial planets lead to a subdivision into a brittle rocky crust, a silicate mantle, and an iron-rich core^1^, the finer details are widely uncertain for Mars. This lack of knowledge hampers our understanding of its geodynamic history, and the formation and evolution of terrestrial planets in general. The three experiments carried out by InSight, and specifically the **S**eismic **E**xperiment for **I**nterior **S**tructure (SEIS), are about to change this.

The study of seismic waves generated by earthquakes has been key to our understanding of the internal structure of the Earth and the Moon. For example, detection of signals reflected at the core–mantle boundary resulted in the determination of the Moon’s core radius. The thickness of the lunar crust was determined using waves converted at the crust–mantle boundary. On Earth, the absence of direct waves from distances beyond 10,000 km, a so-called shadow zone, led to the discovery of Earth’s liquid outer core. Terrestrial seismology nowadays studies 3D fine structure, using 1000 s of seismometers, but on Mars, which was predicted to be seismically active^2^, we still need to answer more basic questions.

Current estimates for the average crustal thickness of Mars range from 30 to >100 km^3,4^. Accurate estimates of crustal thickness are needed to provide an important constraint on the mantle evolution through time along with the formation of the crust. Crustal thickness directly relates to the mantle’s cooling rate, with implications for the style of convection during Mars’ early history (i.e., global mantle overturn, stagnant lid convection, or plate tectonics). Gravity and topography constrain relative crustal thickness variations well, but need at least one tie point to obtain absolute values^3^. Seismology is the only means for this direct measurement.

Mars’ mantle may retain compositional layering from its early evolution, which has been destroyed on Earth by vigorous convection. As Mars lacks plate tectonics, but is of sufficient size to have undergone most of the same differentiation processes as early Earth, its mantle structure may provide important information on planetary formation in general, which is unavailable from the study of the Earth. Distinct compositional layers in the mantle have different elastic properties, making them detectable by seismic waves.

The size of Mars’ core is currently uncertain by at least ± 15%, and it is unknown whether it is entirely liquid or contains a solid inner core, like the Earth’s. The size of the core of a planet determines whether large-scale mantle plumes, which have been postulated to explain Martian volcanoes, can persist over a long period of time, whereas a geodynamo in a liquid core is required to generate a planetary magnetic field. Measurements of strong magnetization in the oldest parts of Mars’ crust indicate that a dynamo once existed, but also that it has vanished. Data on the current state and size of the core, e.g., from waves reflected at the core–mantle boundary or a shadow zone, will help to understand why Mars’ internal magnetic field disappeared.

The estimated number of quakes on Mars per year varies by three orders of magnitude^2^, with an even larger uncertainty for released energy. The amount and distribution of present‐day seismicity, which SEIS will measure for the first time, has implications for the thermal evolution of Mars and the present-day spatial pattern of mantle convection^5^.

## InSight’s heritage and challenges

Seismometers played an important role in early missions to planets and satellites. The first seismometers were sent to the Moon by Ranger 3, 4, and 5 in 1968, and all Apollo missions conducted seismic experiments. Likewise, both Viking Mars landers carried seismometers in 1976. While the Apollo lunar seismic network recorded thousands of moonquakes during its 8 years of operation and shaped our current view of the Moon’s interior^6^, the Viking seismometers were less fortunate. One failed to uncage and the other, located on top of the lander, recorded 19 months of wind noise, but only one candidate marsquake^7^. After these sobering results, seismologists have had to wait for more than 40 years for another chance to hunt for marsquakes.

**Fig. 1:**
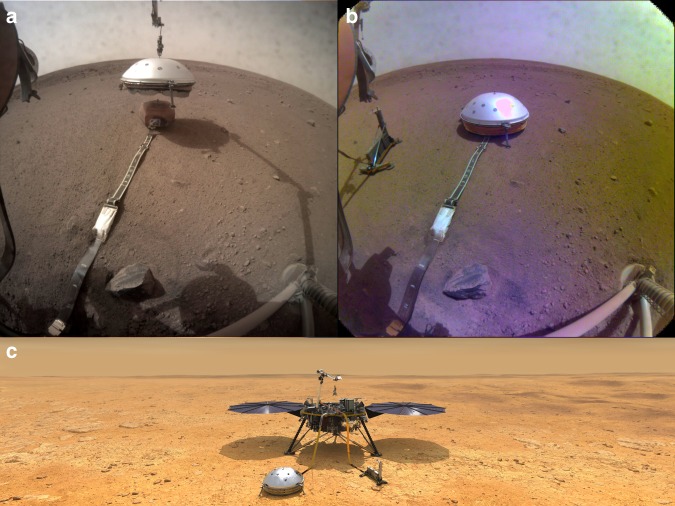
The first seismometer in Elysium Planitia, Mars. **a** Image taken by the south-pointing Instrument Context Camera (ICC) on the InSight lander during the deployment of the WTS to cover SEIS. The sensor assembly itself is contained within the remote warm- enclosure box, the visible orange–brown hexagon. The broadband seismometer itself is furthermore located in an evacuated container to decouple it from the diurnal temperature changes, and shield it from Brownian motion of atmospheric molecules. **b** ICC image of the final deployment, with SEIS beneath the WTS to the right and the Heat flow and Physical Properties Package (HP^3^) sitting to the left. **c** Artist conception of the final deployment situation, including the lander in a panoramic view from the south. Images by NASA/JPL-Caltech.

Compared with installing a seismometer on Earth, the challenges to SEIS were unprecedented: it had to be deployed in a previously unknown spot by a robotic arm and be leveled remotely. All further installation steps, e.g., placing the wind and thermal shield (WTS) right on top of SEIS, also had to be commanded remotely hours in advance, without any chance of direct interaction (Fig. 1). Mars is a hostile environment for a sensitive seismometer, with large temperature swings and episodic winds. Several layers of shielding guard SEIS against adverse influences (Fig. 1). The deployment took more than 2 months, whereas the lunar seismometers were deployed and leveled in less than an hour by astronauts. In addition, SEIS has to deal with the challenges of being the only seismometer on Mars, and thus having to constrain the source of a marsquake and Mars’ velocity structure at the same time.

So far, SEIS, which can detect motions in the nanometer range, measured the lowest seismic noise floor yet recorded anywhere in the solar system^8^ in the period band between 5 and 20 s, which is dominated by oceanic noise on Earth. SEIS is further equipped with a geophysical sensor suite, including wind speed and direction sensors, barometer, thermometer, and magnetometer, which help to identify and remove environmental effects on SEIS. These sensors also produce remarkable science of their own, e.g., by providing continuous meteorological data sampled at a much higher rate than on all previous missions^9^, or by measuring the crustal magnetization at the Martian surface, instead of that from the orbit, for the first time^10^.

## InSight’s first recording of marsquakes and outlook

**Fig. 2:**
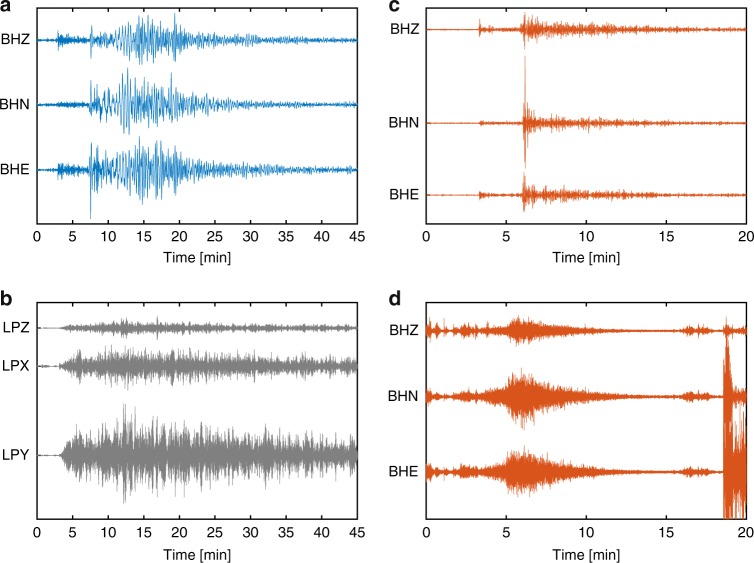
Comparison of seismograms of earthquakes, moonquakes, and marsquakes. **a** Recording of an earthquake in Eastern Turkey of magnitude 6.1, source depth 12 km, at station BFO^15^ on March 8, 2010. The epicentral distance is about 2730 km. Various seismic phases, including P- and S- arrivals and surface waves, are clearly visible. **b** Recording of a shallow moonquake (depth: 50 km) of magnitude 4.07 on January 3, 1975, at Apollo 16. Epicentral distance is about 2690 km, similar to **a**, but the seismogram has a different, spindle-like shape. Scattering within the dry, porous crust masks any clear phase arrivals, and small attenuation leads to a prolonged waveform. **c** Marsquake S0235b recorded by SEIS^13^ on June 26, 2019, at an epicentral distance of about 1540 km, with a magnitude of 3.3. Clear P- and S-arrivals are readily apparent, but surface waves are missing, which could be related to source depth or scattering. **d** Marsquake S0128a recorded by SEIS^13^ on April 7, 2019, with an estimated magnitude between 1.8 and 2.3. This event contains high frequencies, has a spindle-like shape, and no clear phase arrivals. The large amplitudes starting at around 18 min are caused by motion of the robotic arm. This Mars seismograms show a decay time longer than that of Earth and shorter than or comparable to the Moon. This indicates that Mars’ attenuation is stronger than that of the Earth, but weaker than that of the Moon. As attenuation strongly depends on thermal conditions and volatile/water content, such difference may reflect the different environments of the three bodies. Note that the timescale is different in **a** and **b**, and **c** and **d**, respectively. Amplitudes are not to scale between the different subplots.

Mars has proven to be presently seismically active, producing more than 150 quakes within the first 7 months of observation, which form two distinct groups (Fig. 2). This was not anticipated, as both the Earth and the Moon only show one type of waveform. The largest quake recorded thus far had a magnitude M_W_ of 4.0. While Mars was predicted to have less seismicity than the Earth due to the lack of plate tectonics, it seems to surprisingly produce fewer large quakes than would be expected from terrestrial intraplate seismicity^11^. This indicates that small quakes could be more important for the release of seismic energy than on Earth, which was unexpected. For many of the weaker events, it is not yet understood from what kind of source, and where, they originate.Interestingly, two of the largest marsquakes are located near the Cerberus Fossae fracture system more than 1000 km away from InSight^12^, a region of young volcanism suspected to be tectonically active.

By combining seismic signals from closely passing dust devils with data from InSight’s barometer in a previously unfeasible approach, and analyzing the hammering of the heat flow probe HP^3^, SEIS constrained the rigidity of the Martian surface at the landing site, indicating low seismic velocities compatible with unconsolidated sandy material, consistent with local geology^8^. Initial analysis of converted phases in the seismograms of two quakes points to the first crustal layer of 8–11-km thickness with unexpectedly low velocities pointing to highly altered or fractured material^8^. The observation of low amplitudes of S waves in recordings from a specific distance range hints at a low-velocity zone in the Martian mantle, which was predicted by some, but not all, a priori models, and will help to constrain mineralogy^12^. Comparison between Earth, Moon, and Mars seismograms^13^ highlights differences in scattering and attenuation within these three bodies (Fig. 2). The two different types of marsquakes described above correspond to Earth- and Moon-like seismograms (Fig. 2), which can be understood by differences in scattering and attenuation sampled by quakes at different distances from SEIS. The observed waveform variability implies that the Martian interior has at least two distinct layers, with a Moon-like (high scattering and low attenuation) crust covering an Earth-like (low scattering and high attenuation) mantle^8^. While Mars was expected to lie somewhere between Earth and the Moon in terms of these properties, the existence of two different families of marsquakes was surprising.

The next step in the analysis of the rich and unprecedented data from SEIS includes a more in-depth analysis of phases related to the crust to constrain the crustal thickness at the landing site, and continue to further derive seismic velocities and attenuation in the upper mantle. Recordings of larger quakes that excite secondary arrivals, e.g., core reflections, are needed to provide firm constraints on deeper structure.

Even though the above discussion is based on a small number of events, and thus might change with future observations, SEIS has already clearly shown that seismology offers a unique means to unravel the interior structure of Mars. As SEIS remains in good health after more than half of its nominal mission duration, it will probably, like the rovers, exceed all expectations and continue to record marsquakes and initiate new discoveries for the years to come, possibly in collaboration with the SEM seismometer on ESA’s ExoMars 2020 platform^14^. Arrival time data from two seismometers would provide an additional means to triangulate the location of quakes, and not only lead to more precise locations, but also allow locating more quakes. The proposed ExoMars 2020 landing site is at almost 150° distance from InSight and closer to some regions with many observed faults that could be seismically active, e.g., Tharsis and Valles Marineris. In providing a close-up look at the seismic activity of these regions, ExoMars would complement SEIS. InSight could also initiate a welcome comeback of seismometers on planetary missions. A short-period seismometer is proposed for NASA’s Dragonfly mission to Titan, and within the proposed Lunar Geophysical Network, seismometers are viewed as one of the key scientific instruments to be installed. With Titan expected to show seismic activity due to the tidal forcing from Saturn and from impacts, similar to the Moon, a seismometer could constrain the thickness of the ice crust and even detect a high-pressure ice layer at the base of the ocean, with important implications for habitability.
